# Is the Fate of the Internal Mammary Vein in CABG Similar to that of the Saphenous Vein?

**DOI:** 10.21470/1678-9741-2023-0332

**Published:** 2025-05-28

**Authors:** Luis Roberto Palma Dallan, Luis Alberto Oliveira Dallan, Antonio Neves Filho, Omar Asdrubal Vilca Mejia, Luiz Augusto Ferreira Lisboa, Fabio B. Jatene

**Affiliations:** 1 Division of Cardiovascular Surgery, Faculdade de Medicina, Universidade de São Paulo, São Paulo, São Paulo, Brazil. E-mail: luisdallan@gmail.com; 2 Division of Cardiovascular Surgery, Faculdade de Medicina, Universidade de São Paulo, São Paulo, São Paulo, Brazil; 3 Division of Cardiovascular Surgery, Faculdade de Medicina, Universidade de São Paulo, São Paulo, São Paulo, Brazil; 4 Division of Cardiovascular Surgery, Faculdade de Medicina, Universidade de São Paulo, São Paulo, São Paulo, Brazil; 5 Division of Cardiovascular Surgery, Faculdade de Medicina, Universidade de São Paulo, São Paulo, São Paulo, Brazil; 6 Division of Cardiovascular Surgery, Faculdade de Medicina, Universidade de São Paulo, São Paulo, São Paulo, Brazil

Coronary artery bypass grafting (CABG) is an efficient and widely performed procedure in cardiac surgery services around the world. The extensive experience made it possible to use different types of grafts. Among them, we highlight the use of the left internal mammary artery (LIMA) and right internal mammary artery, the radial artery, and the saphenous vein graft (SVG). We recently published a case in which left internal mammary vein (LIMV) was used at CABG, which was patent after six years^[1]^.

## COMMENTS

The favorable effects of the use of LIMA graft to the left anterior descending artery (or LAD) on patient survival and reduction of cardiovascular events are well known^[2]^ and have been extended to other arterial grafts^[3^, ^4^, ^5]^. In patients with multi-arterial lesions, complete myocardial revascularization requires complementation with other grafts and so far, the SVG has been widely used^[6]^.

However, it is recognized that its venous structure can change when submitted to long-term arterial blood pressure regimen. Internal mammary arteries (IMAs) are not usually affected by high blood pressure interference. At the end of 10 years, only 63.3% of venous grafts were considered to be patent^[7]^. The histological comparison between the IMA and the SVG shows a significant difference in their diameters, in the composition of their intima, media, and adventitia layers, in addition to the variation in the presence and amount of elastic and longitudinal fibers. SVGs are known to do adaptive changes when placed in the high-pressure aorta-coronary circulation (“arterialization”), not only intimal thickening but also atherosclerosis. These anatomical variations justify the superiority of IMA graft over SVG^[8]^. However, nothing is known about the behavior of mammary vein as a graft, especially if subjected for a long time to systemic pressure conditions.

In the case reported^[1]^, the patient was submitted to a coronary angiogram six years after CABG that showed the LIMV patent in good condition, whereas the SVG presented 60% of obstruction on its path. A coronary angiotomographic study, carried out on the same patient 16 years after CABG, did not allow visualization of the LIMV graft, which questions its patency.

Therefore, the question remains: Will the mammary vein have in the long term the same fate as grafts with the saphenous vein?

Undoubtedly, using both IMAs to graft the main coronary arteries in young patients remains the gold standard.

We believe that the use of LIMV as a complementary graft in CABG could be considered in those rare symptomatic patients who have a complex coronary disease and do not have traditional grafts available to harvest, including those with peripheral vascular disease, fistula for dialysis, and venous insufficiency.

Additional studies are needed to establish the validity of its long-term use.
